# Supplementary material for: Integration of partially observed multimodal and multiscale neural signals for estimating a neural circuit using dynamic causal modeling
Source: PLoS Comput Biol. 2024 Dec 23;20(12):e1012655. doi: 10.1371/journal.pcbi.1012655 (PMC11706407; doi:10.1371/journal.pcbi.1012655)
Supplement: S1 Text — The supplementary document explains the methodologies for extracting and analyzing calcium imaging signals from the barrel cortex, which were used to create a ground truth system. (PDF) [file pcbi.1012655.s001.pdf]

Supporting Information

Integration of Partially Observed Multimodal and Multiscale Neural Signals for Estimating a Neural Circuit Using Dynamic Causal Modeling

Jiyoung Kang[1,2] and Hae-Jeong Park[2,3,4,5]*

[1]Department of Scientific Computing, Pukyong National University, Busan, Republic of Korea

[2]Center for Systems and Translational Brain Sciences, Institute of Human Complexity and Systems Science, Yonsei University, Seoul, Republic of Korea

[3]Graduate School of Medical Science, Brain Korea 21 Project, Department of Nuclear Medicine, Psychiatry, Yonsei University College of Medicine, Seoul, Republic of Korea

[4]Department of Cognitive Science, Yonsei University, Seoul, Republic of Korea

[5]Brain Research Institute, Institute for Innovation in Digital Healthcare, Yonsei University College of Medicine, Seoul, Republic of Korea

*Email: parkhj@yonsei.ac.kr

CaI signal extraction

We used CaI signals of the barrel cortex of a mutated mouse (animal id: an194672) during a single whisker object localization task [1], which are publicly available (https://crcns.org/data-sets/ssc/ssc-2/about-ssc-2). The mouse was mutated (*Emx1-Cre* × *LSL-H2B-mCherry*) to express a red fluorescent protein (mCherry) in the nuclei of cortical glutamatergic excitatory neurons. To determine the concentration of cytosolic calcium ions in neurons, all neurons in and around the mouse's principal column were infected with AAV2/1 syn-GCaMP6s [2]. CaI data were sampled at 7 Hz with 600 × 600 μm^2 (512 × 512 pixels) at three planes separated by 15 m in depth. To define regions in the barrel cortex and to extract neurons that have similar patterns, we performed independent component analysis (ICA) for the signals obtained from excitatory and inhibitory neurons (Figure S1A) [3].

In the ICA, a maximum of five components were assumed for excitatory and inhibitory neurons based on the results of single value decomposition (SVD) (Figures S1B and S1C). Among the five components, we selected two excitatory and one inhibitory independent components (ICs) that showed fast response from the stimulus (Figure S1C), according to the conventional configuration of

a cortical column in the Jansen-Rit model [4, 5]. To account for the effects of the neurons' spatial distributions, we divided the entire area into four regions. We labeled each neuron with the corresponding IC, and reconstructed IC signals from selected cells for each region, except for cells with a low weight (0.07) for IC signals. We adjusted the region (denoted column in the current study) boundaries to produce similar patterns for each selected component, which we measured using the sum of root mean square deviation (RMSD) between two regions,

$$RMSD(r1, r2; j) = \sqrt{\frac{1}{T}\sum_{i=1}^{T}\left(IC_{r1,j}(t) - IC_{r2,j}(t)\right)},$$

Where r1 and r2 are the indexes of the region, and j represents the index of IC. For each region, final IC signals were obtained from 6-15 excitatory and 2-10 inhibitory neurons. Finally, we used a low pass filter of 0.1 Hz to reduce noise (Figure S1D).

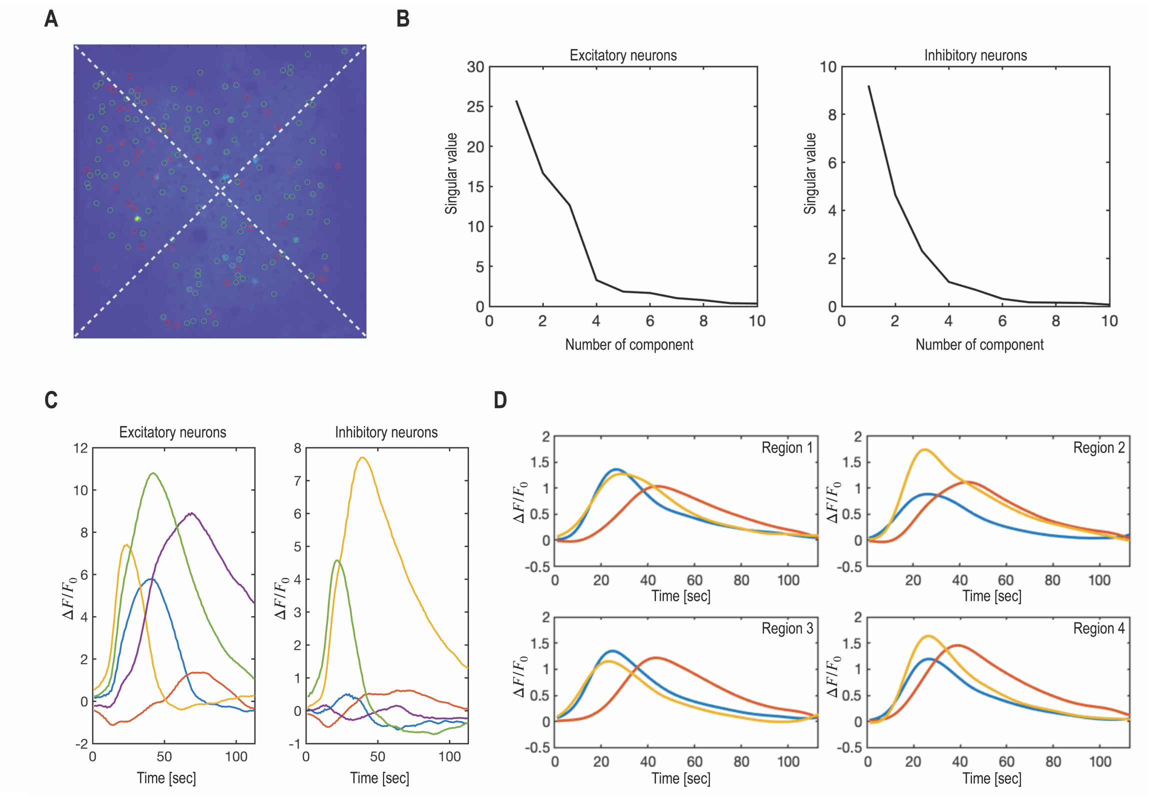

Figure S1. Assignment of CaI signals for each region. (A) Spatial distributions of the neurons are displayed with green (excitatory neuron) and red (inhibitory neuron) circles. The white dotted line separates regions. (B-C) Based on the results of SVD, we selected five ICs for excitatory and inhibitory neurons, which are displayed in (C). We further selected two excitatory and one inhibitory ICs that showed fast response from the stimulus; components shown with blue and yellow colors and

components shown with green color were selected for excitatory and inhibitory neurons. (D) We adjusted the boundaries of the regions to contain neurons with similar signals. Two excitatory and one inhibitory signals are displayed with blue, red, and yellow colors.

References

1. Peron SP, Freeman J, Iyer V, Guo C, Svoboda K. A Cellular Resolution Map of Barrel Cortex Activity during Tactile Behavior. Neuron. 2015;86(3):783-99. Epub 2015/04/29. doi: 10.1016/j.neuron.2015.03.027. PubMed PMID: 25913859.
2. Chen T-W, Wardill TJ, Sun Y, Pulver SR, Renninger SL, Baohan A, et al. Ultrasensitive fluorescent proteins for imaging neuronal activity. Nature. 2013;499(7458):295-300. doi: 10.1038/nature12354.
3. Jung K, Kang J, Chung S, Park HJ. Dynamic causal modeling for calcium imaging: Exploration of differential effective connectivity for sensory processing in a barrel cortical column. Neuroimage. 2019;201:116008. Epub 2019/07/14. doi: 10.1016/j.neuroimage.2019.116008. PubMed PMID: 31301360.
4. Jansen BH, Rit VG. Electroencephalogram and visual evoked potential generation in a mathematical model of coupled cortical columns. Biol Cybern. 1995;73(4):357-66. Epub 1995/09/01. PubMed PMID: 7578475.
5. Moran R, Pinotsis DA, Friston K. Neural masses and fields in dynamic causal modeling. Frontiers in computational neuroscience. 2013;7:57. Epub 2013/06/12. doi: 10.3389/fncom.2013.00057. PubMed PMID: 23755005; PubMed Central PMCID: PMCPMC3664834.
