# Supplementary material for: Integration of partially observed multimodal and multiscale neural signals for estimating a neural circuit using dynamic causal modeling
Source: PLoS Comput Biol. 2024 Dec 23;20(12):e1012655. doi: 10.1371/journal.pcbi.1012655 (PMC11706407; doi:10.1371/journal.pcbi.1012655)
Supplement: Attachment — Submitted filename: responses_to_reivewers.pdf [file pcbi.1012655.s002.pdf]

Response to Editor and Reviewers

We sincerely appreciate all the reviewers' valuable and insightful comments. In this revision, we have made a concerted effort to address each reviewer's suggestions comprehensively.

Below, we provide detailed point-by-point responses to the comments, presented in italics.

Reviewer #1:
This study introduces a dynamic causal modeling (DCM) method to address the challenge of integrating unevenly observed, multiscale signals within a larger-scale neural circuit. This is achieved by employing a shared neural state model and observation models for different imaging modalities. The authors propose a method that reciprocally integrates local and global information from different modalities to infer the target circuit.

The proposed reciprocal and iterative technique substantially improves the estimation of local and global connectivity compared to traditional one-step estimation approaches, which typically rely on less informative priors for unobserved data.

This study raises important questions, particularly relevant to the DCM community, where causal inference is widely applied across different domains. The results are interesting, but the paper needs more clarity regarding what has been accomplished. Specifically, the introduction and abstract are written in a very generic manner, which raises issues related to what exactly has been done. From the introduction, I was left with the impression that one could infer calcium imaging (CaI) from fMRI data, which seems misleading!!

Response
First, we sincerely appreciate your constructive comments and suggestions. In response, we have worked to clarify the manuscript and enhance the quality of the paper. Notably, we have rephrased most of the Abstract and Introduction. Please see the revised manuscript. Below, we address each of your comments in detail.

Q1) If local and global information informs each other, should there not be a sequential relationship between them, or can they be decoupled? Why are they not inferred simultaneously from the data?

Response
Thank you for your insightful question. This paper indeed argues that local and global information are interdependent and should complement each other in parameter estimation. You raise an important consideration regarding the estimation sequence—whether parameters should be estimated sequentially or simultaneously. Although simultaneous estimation is often ideal, it can pose challenges when signals are sparsely sampled across regions and scales, or when a large number of parameters are required for a comprehensive network model.
For partially observed data on local circuits, we adopted a strategy that uses parameter estimates from observed local circuits as priors for unobserved regions in the global circuit, applied sequentially.

Simultaneous estimation of all parameters across all data would significantly increase complexity, making a sequential approach more practical.

Another important aspect of the current method is its formulation strategy. We used exponential parameterization for each neural property, separating a reference value from its modulation parameter. Here, the reference values act as hyperparameters, while modulation parameters function as conventional parameters. Since both need to be estimated, the optimization process typically proceeds sequentially, first selecting optimal hyperparameters, then fine-tuning with the modulation parameters.

An additional advantage of estimating reference values first is that it enables greater control over inter-regional consistency. We applied a common reference value for each neural property estimated at the local circuit level across different regions, which constrains local circuit parameters to remain consistent during global circuit estimation. By assigning these priors (common reference values) to regions without observed signals—based on estimates from regions with observed signals during global circuit estimation—we maintained parameter estimates within a consistent range of neural properties across regions, allowing for slight regional variations in the final DCM. Linking neural properties in this way reduces the search space, enhancing computational efficiency in terms of both speed and memory, which is crucial in Bayesian approaches.

Furthermore, the large number of parameters can present challenges, especially in larger systems, as demonstrated in our study. A high parameter count increases the risk of degeneracy, where multiple plausible solutions could fit the limited data, complicating the identification of biologically meaningful solutions.

To address these challenges, we propose a sequential iterative estimation procedure as an effective solution. We have incorporated these points into the Discussion section for further clarification.

Page 33

"Theoretically, there is no inherent prioritization between estimating local intra-regional and global inter-regional parameters; instead, iterative estimation facilitates convergence by allowing these parameter sets to inform each other reciprocally. Practically, however, it is preferable to first estimate low-level biological features, which then serve as a basis for establishing global parameters. We assumed that local microscopic biological properties are generally consistent across different brain regions compared to the more variable inter-regional global parameters. Based on this assumption, we assigned priors to parameters for regions without observed signals using parameter estimates from regions with observed signals, thereby enhancing the efficiency of the entire parameter estimation process. Additionally, under this assumption, we used BAYESopt to search for reference values for local properties. By using a single reference value for each biological property across all local regions, this approach constrains parameter estimates for these properties within a similar range across regions, even after final DCM fine-tuning. Conversely, suppose we do not use a single reference value but instead use DCM with a wide prior variance for each biological property in each region. In that case, we cannot ensure that biological properties remain within a similar range across local regions."

Q2) In the introduction, the phrase "combining unevenly observed multimodal signals" is unclear—how does this apply? In terms of dimension? It wasn't clear from the introduction what is meant by multimodal multiscale inference. As a reader, should I expect to see the inference of CaI from VSDI or fMRI? Does this

involve all the activity of CaI or only certain signals? Combining them could indeed improve model evidence, but the introduction gives the impression that one can infer CaI from EEG/fMRI or, more generally, infer fMRI from EEG/MEG, which seems overly ambitious. If this works, does it mean one can generate recordings that we currently don't know how to measure?

Response

We apologize for any confusion caused by the unclear writing in the Introduction. As you correctly noted, the current method does not infer missing signals from other signals; rather, all available signals are used together to estimate the model parameters of a single neural state (circuit) model. We have thoroughly revised the Introduction to clarify these points and prevent any potential misinterpretations. Thank you again for your valuable feedback, and please refer to the revised Introduction for these updates.

Q3) My main concern is: if EEG and fMRI data are combined into a single system (shared neural state model), then the system must be dynamically rich and multiscale (i.e., involve slow and fast timescales, up/down states, and limit cycles). How is this feasible? Wouldn't you need different mappings (gain matrices) from source to sensors for each modality? Are these mappings known? How are the modalities coupled in the state space? For example, the Epileptor model offers a taxonomy of seizures (Jirsa et al., Brain 2014, 10.1093/brain/awu133), but this requires strong a priori knowledge about the combined modalities. It would be very helpful to formulate the problem in a state-space framework and clearly define what is known, what is observed, and what is inferred (with dimensions included).

Response

Thank you for these insightful questions. As you correctly pointed out, integrating multimodal data at different spatial and temporal scales requires a system with rich dynamics and multiscale characteristics. Although we did not consider EEG and fMRI at a macroscopic level with whole-brain coverage, we recognize that your question highlights the need for a neural state model that captures diverse aspects relevant to both EEG and fMRI signals.

In our current framework for the animal study, we employ a conventional neural state dynamic system with different mappings for each modality. The neural state model used in the current study is not new and has often been used for diverse applications with independent measurements of CaI, VSDI or fMRI, without combinations. Signals from each modality are represented through observation models that map neuronal population activity to observable signals. These mappings rely on established a priori knowledge from previous studies, with methodologies specific to each modality. For example, we draw on key studies for calcium imaging (CaI) [1, 2], voltage-sensitive dye imaging (VSDI) [3, 4], and blood-oxygen-level-dependent (BOLD) signals [5, 6]. Thus, as you commented, different source to sensor mapping according to each modality is used together to compliment model parameter estimation. In summary, to estimate a single neural circuit at a specific brain state, we have combined multimodal signals using modality-specific source to sensor mapping.

This approach differs from models like the Epileptor (Jirsa et al., 2014), which reproduce various neural circuit states through distinct parameter configurations. Unlike the Jirsa model, which focuses on diverse neural state dynamics representing a wide range of possible system states, our study focuses on estimating system parameters at a specific brain state with sparsely observed data—data that might only reflect a

specific state of the system. When data from a range of system states become available, the neural state model may indeed need to be expanded to accommodate and explain all these diverse states, a challenge that remains beyond the scope of our current study.
While we aimed to clearly explain our model and experiment in the manuscript, we recognize the need for further clarification. In response to your recommendation, we have added a paragraph to the Methods section to specify what should be inferred, and we have slighly revised the paragraph in the Discussion to address these issues.

Page 10

"In summary, the goal of the current framework is to estimate unknown model parameters—both neural state model parameters (Table 1) and observation model parameters (Table 2)—using observed signals, a combination of multimodal data $[Y_{CaI}, Y_{VSDI}, Y_{BOLD}]$ from a single neural system. All parameters to be estimated in this study are detailed in Tables 1 and 2."

Page 33

"Furthermore, while our current framework is built on a convolution-based model, its versatility allows for extension to other types of neural state models. An example of this adaptability can be seen in the conductance model demonstrated by Shaw, Muthukumaraswamy (41). Additionally, it can be adapted for a multi-state neural dynamics model, as explored in the context of diverse seizure states [42]. This flexibility suggests that our approach could be refined and expanded in future research to accommodate a broader range of neural dynamics and conditions."

Q4) For Figure 2, it would be helpful to show true versus estimated effective connectivity.

Response
Thank you for this suggestion. In Figure 2, we present the estimated results of the foundational model used in this experiment, which is based on experimental data. As a result, we do not have access to the true effective connectivity for comparison. Instead, the estimated parameters from the foundational model are used to construct diverse virtual systems for testing the proposed framework. We have clarified this point in the revised manuscript.

Page 16

"Note that these parameters were estimated from experimental data obtained from a mouse. Although the accuracy of the model parameters has not been validated, we considered these parameters as a ground truth reference for subsequent virtual experiments by simulating diverse signals."

Q5) For Table 3, it would be beneficial to describe the estimations—if they are the same, perhaps in the caption, but if they differ, include a column for each experiment.

Response

First, we apologize for any confusion caused by the table numbering. Regarding your comment on the updated Table 4, “Summary of Experiments,” we’re not entirely certain if we’ve fully understood your suggestion. Based on our interpretation, we believe you are inquiring about the consistency of the estimation methods and strategies across experiments. We confirm that the methods and strategies are indeed consistent across all experiments, and we have clarified this in the Table caption.

Q6) How strong is the prior, and what is the gain of information from prior to posterior? For instance, Hashemi et al. introduced a fully Bayesian method to examine the influence of priors on estimation (https://doi.org/10.1371/journal.pcbi.1009129).

Response

Thank you for your constructive comment and suggestion. Following your recommendation, we evaluated posterior shrinkage for Experiment 5. Although our parameter estimation method, which uses variational Bayes, differs from the MCMC-based approach used in Hashemi et al., we calculated the shrinkage of the posterior for each parameter as follows:

$$\rho_i = 1 - \frac{\sigma_{i,post}^2}{\sigma_{i,prior}^2}.$$

where $\sigma_{i,prior}^2$ and $\sigma_{i,post}^2$ represent the variances of prior and posterior distributions.

We compared intra-regional effective connectivity (A_{intra}) and inter-regional effective connectivity (A_{inter}) for Experiment 5. The results, shown in Figure 10, demonstrate that the iterative method increased precision relative to the priors and also reduced the posterior variance more effectively for A_{intra} values than for A_{inter} values. This indicates that our approach can extract more information from the data by iteratively updating priors, particularly enhancing parameter estimation for local circuits. We have added paragraphs detailing this evaluation to the Results section. Thank you again for this constructive suggestion.

Page 31

“To evaluate how effectively our estimation methods refined parameter estimates by reducing uncertainty, we calculated the posterior shrinkage, as described in [31], defined by: defined by:

$$\rho_i = 1 - \frac{\sigma_{i,post}^2}{\sigma_{i,prior}^2},$$

where, $\sigma_{i,prior}^2$ and $\sigma_{i,post}^2$ represent the variance of prior and posterior distribution of parameter i.

We compared the posterior shrinkage of effective connectivity between the iterative and one-step estimation methods for both intra- and inter-regional connectivity. The results showed a significant reduction in variance from prior to posterior estimates in the iterative estimation process, particularly for intra-regional connectivity, as indicated by higher ρi values in the blue box plots compared to the red box plots in Figure 10. In contrast, the one-step estimation method showed relatively less reduction in posterior variance for both intra- and inter-regional connectivity. These findings suggest that the iterative method more effectively utilized information from the data. We further compared the shrinkages of posterior of effective connectivity

between intra- and inter- regional connectivity in experiment 5. The results demonstrate a significant reduction in variance from prior to posterior estimates in iterative estimation, particularly in the intra-regional connectivity estimates as shown by the higher ρi values in blue box plots compared to red as shown in Figure 10. On the contrary, in one-step estimation, both intra- and inter- regional effective connectivity were not significantly reduced as in those of the iterative estimation. These findings suggest the effectiveness of our methods in extracting more information from the data.

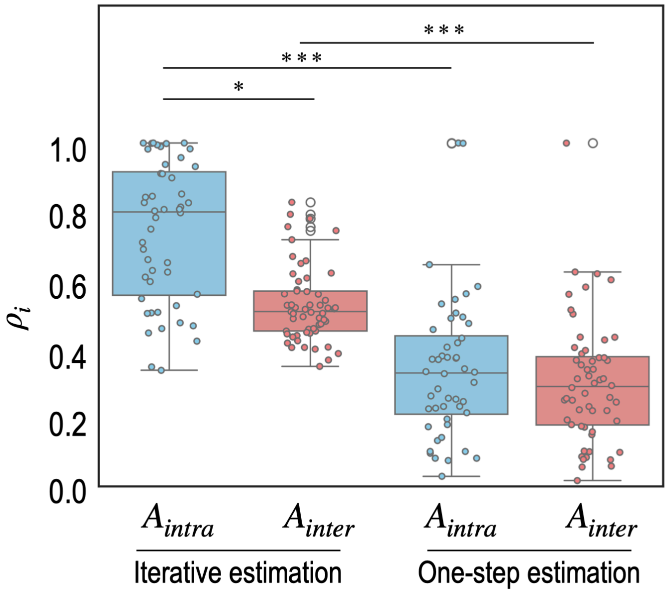

Figure 10. Posterior shrinkages from prior distributions for each effective connectivity in experiment 5. Posterior shrinkages (ρi) of each parameter i from prior distributions in iterative estimation (left) and one-step estimation (right) are displayed, respectively. Both panels use blue box plots for intra-regional (Aintra) and red for inter-regional (Ainter) connectivity, with significant differences marked by three asterisks (* and *** represents $p < 0.01$ and $p < 0.0001$, respectively). Statistical significance was assessed using the Kruskal-Wallis test since the groups have different variances, violating the homogeneity of variance assumption required for one-way ANOVA.

Q7) What are the limitations of the study, and under what conditions does the algorithm fail (in-silico)? For example, in terms of parameter/variable dimensions? SEEG signals are sparse, and inference from them is a formidable challenge. The approach used in this paper could be very helpful in informing non-identifiable parameters.

Response

Thank you for this valuable suggestion. While we have outlined the limitations of the current study, we have expanded on the limitation in the Discussion section. We consider that the main challenge is not the failure to reach a solution, but rather ensuring the validity of the solution in representing real biological

systems. Achieving this would require a more accurate model with additional biological constraints. Please refer to the updated Discussion section for further details.

We also agree that our framework has the potential to assist in circuit estimation using SEEG, where sparsely sampled signals make inference particularly challenging.

Page 35

"Additionally, the simplification that local neural circuits are uniform across different brain areas may introduce inaccuracies, especially within large and heterogeneous neural systems. Furthermore, given the higher number of parameters relative to observational data, particularly in the nonlinear system, there is a risk that different effective connectivity configurations could yield similar observational outcomes, introducing potential degeneracy, as discussed in prior studies [39, 40]. Thus, the primary challenge is not reaching an optimal solution but ensuring that this solution is valid and accurately represents real biological systems. Addressing this would require a more precise model with additional biological constraints. These limitations pertain more to the selection of an appropriate model than to the parameter optimization approach addressed in this study."

"For instance, this method could be applied using combinations of electrocorticogram (ECoG) or stereo-electroencephalography (SEEG) with EEG or MEG signals for large-scale whole-brain circuit analysis, particularly in the study of epilepsy."

Q8) Lastly, is there any investigation or comment on the degeneracy (or non-identifiability) of effective connectivity?

Response

Thank you for raising this important concern about the degeneracy of effective connectivity. Given the nonlinear nature of the system and the higher number of parameters relative to observed data, it is indeed possible for different effective connectivity configurations to produce similar observational signals, leading to degeneracy, as noted in previous studies [2, 3]. We have addressed this issue in the Discussion section.

Page 35

"Furthermore, given the higher number of parameters relative to observational data, particularly in the nonlinear system, there is a risk that different effective connectivity configurations could yield similar observational outcomes, introducing potential degeneracy, as discussed in prior studies [39, 40]."

We appreciate all of your informative comments.

Reviewer #2:

This study addressed an ambitious modelling challenge: how to infer the common neural dynamics that give rise to neuroimaging measurements at different spatial and temporal scales, specifically calcium imaging, voltage-sensitive dye imaging and BOLD fMRI. The novelty of the authors' solution is 1) the pairing of a single neural model with modality-specific observation models, and 2) implementation of Bayesian model inversion methods, specifically the initialization of parameters using a scheme called Bayesian Optimization, and the design of an efficient scheme for iteratively estimating local and global parameters. This is an excellent piece of work and a strong contribution to the field. I just have a single query / suggestion for the authors, that relates to their novel scheme for model parameter estimation (3.1.2).

The authors began model estimation using Bayesian Optimization (BAYESopt) to select suitable priors for subsequent estimation using the typical Variational Laplace (VL) scheme in DCM. It took me a bit of time to understand this. I suggest ***adding a paragraph to the methods section introducing Bayesian Optimization and clarifying the distinction between this and the standard VL scheme (which of course is also a scheme for Bayesian optimization)****. In particular, could the authors explain whether BAYESopt offer some advantage over simply relaxing the prior variances to make them uninformative and using the VL scheme?*

Response

We would like to thank you for your positive feedback and very detailed comments. We apologize for any confusion caused by the organization of the previous manuscript. In response to your suggestion, we have added Section 3.1.3 in the Methods to clarify the distinction between Bayesian Optimization (BAYESopt) and the Variational Laplace (VL) scheme used in DCM.

For example, we now include the following explanation on Page 14:
"Among Bayesian optimization algorithms, we implemented a Gaussian process model as the surrogate model [28] and used an expected-improvement-per-second-plus function for the acquisition function [29, 30]. This approach provides a point estimate, while DCMopt, using the variational Laplace scheme, estimates model parameters in terms of posterior distribution, as explained in the previous section."

Thank you also for your suggestion about comparing Bayesian Optimization with a VL scheme using non-informative priors. We did test the approach of relaxing prior variances to reduce constraint; however, we found that this did not increase the range of parameter exploration and led to unsatisfactory results. The primary issue is that our model assumes consistency (or only slight variations) in biological properties across brain regions, especially in local properties. Simply relaxing the priors of neural properties at each region disrupts this constraint, resulting in inconsistent parameter ranges across regions. By using Bayesian Optimization to determine shared reference values for local neural properties, we maintain consistency while allowing for slight variations (a "jitter") using modulation parameter in the final DCM estimation.
Once again, we appreciate your helpful comments, and we have clarified these points in the updated Discussion section.

“**Bayesian optimization and DCM for model parameter estimation**

Due to the exponential parameterization, model parameter estimation requires determining both a reference value and its modulation parameter. The reference value is first established, and then the modulation parameter is fine-tuned, rather than estimating both simultaneously. In a conventional optimization setting, the reference value functions as a hyperparameter. To enhance the efficiency of parameter estimation, we employed Bayesian optimization (referred to as BAYESopt, distinct from DCM in Eq. 18) [28] to determine the reference values. These reference values then serve as priors for fine-tuning DCM parameter estimation (Eq. 18), facilitating an effective fit to the experimental data. …”

Page 33

“Theoretically, there is no inherent prioritization between estimating local intra-regional and global inter-regional parameters; instead, iterative estimation facilitates convergence by allowing these parameter sets to inform each other reciprocally. Practically, however, it is preferable to first estimate low-level biological features, which then serve as a basis for establishing global parameters. We assumed that local microscopic biological properties are generally consistent across different brain regions compared to the more variable inter-regional global parameters. Based on this assumption, we assigned priors to parameters for regions without observed signals using parameter estimates from regions with observed signals, thereby enhancing the efficiency of the entire parameter estimation process. Additionally, under this assumption, we used BAYESopt to search for reference values for local properties. By using a single reference value for each biological property across all local regions, this approach constrains parameter estimates for these properties within a similar range across regions, even after final DCM fine-tuning. Conversely, suppose we do not use a single reference value but instead use DCM with a wide prior variance for each biological property in each region. In that case, we cannot ensure that biological properties remain within a similar range across local regions.”

References

1. Penny WD. Comparing dynamic causal models using AIC, BIC and free energy. Neuroimage. 2012;59(1):319-30. Epub 20110727. doi: 10.1016/j.neuroimage.2011.07.039. PubMed PMID: 21864690; PubMed Central PMCID: PMCPMC3200437.

2. Edelman GM, Gally JA. Degeneracy and complexity in biological systems. Proc Natl Acad Sci U S A. 2001;98(24):13763-8. Epub 20011106. doi: 10.1073/pnas.231499798. PubMed PMID: 11698650; PubMed Central PMCID: PMCPMC61115.

3. Marder E, Goaillard J-M. Variability, compensation and homeostasis in neuron and network function. Nature Reviews Neuroscience. 2006;7(7):563-74. doi: 10.1038/nrn1949.
